# Hydroxytyrosol Bioavailability: Unraveling Influencing Factors and Optimization Strategies for Dietary Supplements

**DOI:** 10.3390/nu17182937

**Published:** 2025-09-12

**Authors:** Marta Jordán, Natalia García-Acosta, José Luis Espartero, Luis Goya, Raquel Mateos

**Affiliations:** 1 Department of Metabolism and Nutrition (DMN), Institute of Food Science Technology and Nutrition (ICTAN), Spanish National Research Council (CSIC), C/José Antonio Novais 6, 28040 Madrid, Spain; mjordan@ictan.csic.es (M.J.); n.garcia@ictan.csic.es (N.G.-A.); luisgoya@ictan.csic.es (L.G.); 2 Department of Biochemistry and Molecular Biology, Faculty of Chemistry, Complutense University of Madrid, 28040 Madrid, Spain; 3 Department of Biochemistry and Molecular Biology, Faculty of Pharmacy, Complutense University of Madrid, 28040 Madrid, Spain; 4 Department of Organic and Pharmaceutical Chemistry, Faculty of Pharmacy, University of Seville, 41012 Sevilla, Spain; jles@us.es; 5 CIBER de Diabetes y Enfermedades Metabólicas Asociadas (CIBERDEM), Instituto de Salud Carlos III (ISCIII), 28029 Madrid, Spain

**Keywords:** hydroxytyrosol, bioavailability, extra virgin olive oil, dietary supplements, food matrix, delivery systems, encapsulation, metabolism, phenolipids, gut microbiota

## Abstract

Hydroxytyrosol (HT) is a major phenolic compound in olives and extra virgin olive oil (EVOO), known for its antioxidant, anti-inflammatory, and cardiometabolic properties. The European Food Safety Authority (EFSA) has approved a health claim for the protection of LDL particles from oxidative damage only when HT is consumed within EVOO, which limits its direct use in supplements or functional foods. Since its biological effects depend on absorption, distribution, metabolism, and excretion (ADME), understanding how formulation and delivery strategies influence bioavailability is essential. HT is mainly present as secoiridoid derivatives in EVOO, whereas in supplements, it often appears in its free form, potentially affecting its metabolic fate. This review summarizes human studies on HT bioavailability from EVOO, isolated supplements, and enriched foods, and examines how matrix type, chemical modifications of HT, and advanced delivery systems, such as emulsions, encapsulation, and vesicular carriers, modulate absorption and metabolism. The gut microbiota is highlighted as an emerging factor in HT biotransformation, although its role remains underexplored. Further well-designed human studies are needed to guide the development of nutraceutical formulations capable of replicating the health benefits of EVOO beyond its natural matrix.

## 1. Introduction

Hydroxytyrosol (3,4-dihydroxyphenylethanol; HT) is a phenolic compound of considerable biological and pharmacological interest. It is widely recognized as one of the most powerful natural antioxidants found in olive-derived products [1,2]. Structurally, HT is a small amphipathic molecule characterized by a hydroxylated phenolic ring. The presence of this phenolic group confers strong redox properties and a remarkable capacity to scavenge free radicals [3]. These characteristics not only enhance its antioxidant potential but also underlie a broad spectrum of bioactivities, including anti-inflammatory, cardioprotective, and neuroprotective effects [4]. HT is considered a key contributor to the health benefits traditionally associated with the Mediterranean Diet, whose foundation is deeply rooted in the consumption of extra virgin olive oil (EVOO), often described as “liquid gold.” This oil serves as the main source of hydroxytyrosol, which exists primarily in the form of secoiridoid derivatives, along with smaller amounts of its free form and the acetylated derivative hydroxytyrosyl acetate [5]. The polyphenols present in olive oil have received increasing scientific attention, particularly since the European Food Safety Authority (EFSA) approved the health claim that “olive oil polyphenols help to protect low-density lipoproteins (LDL) from oxidative damage” [6], an effect attributed to their antioxidant activity. However, the fact that HT and its derivatives are found almost exclusively in olive-derived products limits their consumption in regions far from the Mediterranean basin. In this context, the expanding dietary supplements market offers an opportunity to increase HT consumption among populations that do not traditionally include EVOO or related products, such as table olives, in their diet [7]. While supplementation improves accessibility, the health effects of isolated HT remain less well understood, particularly since the EFSA health claim applies only when these phenols are consumed within the EVOO matrix [6]. This restriction reflects the fact that the EFSA opinion was based on clinical studies using EVOO as the delivery matrix. The lipid environment and minor constituents of EVOO contribute to the stability and absorption of HT, and potential synergistic effects cannot be assumed for isolated supplements. Therefore, additional well-designed human studies with isolated HT are required before the health claim can be extrapolated to other formulations. Nevertheless, following its approval as a novel food in the European Union, after a safety evaluation [8], HT can now be incorporated into dietary supplements or used as an ingredient in other foods. As a result, this compound has attracted considerable interest in the nutraceutical field.

From a physiological perspective, the effectiveness of HT depends not only on its residence time in systemic circulation but also on its metabolic fate, as it is metabolized into other compounds with variable excretion rates and biological activities. In other words, its efficacy is intrinsically linked to its bioavailability and biotransformation. Despite its promising bioactivities, HT faces several challenges that can limit its therapeutic potential, including rapid metabolism and excretion [9], low solubility in oil [10], and interactions with the gut microbiota [11,12]. Overcoming these limitations is critical to maximizing its health benefits, particularly in the context of nutraceuticals and dietary supplements.

Most human studies on HT bioavailability have been conducted using olive oil as the delivery matrix [13,14,15,16], while fewer studies have investigated HT-rich extracts obtained from olive pomace, leaves or other by-products [17]. In this regard, studies employing isolated HT or chemically modified derivatives could provide more consistent data on its absorption, metabolism and physiological effects.

The aim of this review is to provide a comprehensive overview of HT bioavailability in humans, with a particular focus on the factors that influence it and the strategies that could enhance it in dietary supplements. This work seeks to integrate current knowledge, highlight key modifying factors, and identify potential approaches to optimize HT absorption and efficacy, thereby offering an unified perspective on this topic.

## 2. Materials and Methods

As a starting point for this non-systematic literature review, a report [8] describing safety of hydroxytyrosol as a novel food pursuant by was used. This report included 44 references, which were initially evaluated as potential sources of information. Ultimately, 8 references were selected for their focus on HT bioavailability in humans.

Subsequently, a comprehensive search was conducted in the Web of Science Core Collection database, selecting both reviews and original research articles, using the following terms: hydroxytyrosol AND human AND (bioavailability OR metabolic OR metabolite OR metabolism OR delivery system OR vehiculation OR matrix OR transport OR vehicle OR administration OR emulsion OR capsule OR liposome OR trial OR assay OR nutraceutical OR supplement OR absorption OR ADME OR excretion OR distribution OR pharmacokinetics OR gut OR microbiota OR colonic) for the topic. This search yielded 458 results from publications dated between 2016 and July 2025.

A more refined search was then performed by excluding specific topics unrelated to bioavailability or nutraceutical applications. This was achieved by applying the following exclusion criteria to the title field: NOT cancer NOT chondrocyte NOT signal NOT diabetic NOT conversion NOT therapy NOT toxic NOT catalyst NOT inflammation. After applying these filters, 385 publications remained.

Subsequently, studies were then categorized by species using title and topic filters. For human studies, “AND human” was applied, while “AND (mice OR rats)” was used to identify animal models. This resulted in 34 human studies and 110 studies conducted in mice and rats.

Considering the limited number of human studies, the search was then expanded to include publications from 2010 onwards, resulting in 56 additional articles, of which 8 duplicates were removed.

A positive selection process was then carried out by screening titles and abstracts, classifying the remaining studies into the following categories: general concepts and bioavailability (17), human bioavailability (21), HT delivery methods (21) and gut microbiota (22).

Finally, 10 additional cross-references were included for their high relevance to the review objectives, resulting in a final selection of 91 references considered for this review.

## 3. Results and Discussion

### 3.1. Hydroxytyrosol Bioavailability from Olive Oil

Virgin olive oil (VOO) is the primary dietary source of HT and serves as a well-established model to investigate its absorption, distribution, metabolism, and excretion (ADME). However, it should be noted that in VOO, HT is predominantly present as secoiridoid derivatives, which may influence its bioavailability in humans compared to dietary supplements, where HT is often provided in its free form. This is particularly relevant since HT absorption is strongly modulated by the surrounding food matrix [18]. The lipid-rich environment of VOO promotes the solubilization and intestinal uptake of HT and its secoiridoid derivatives, with human studies reporting peak plasma concentrations (Cmax) within 30–60 min after ingestion, mainly as sulfated and glucuronidated conjugates [19,20]. This pharmacokinetic profile has been consistently observed in multiple clinical trials (Table 1).

A notable study reported HT peaking at approximately 32 min after ingestion of 25 mL of VOO, with all identified metabolites reaching maximum plasma levels within one hour [21]. Similarly, another trial [22] using alperujo-derived polyphenols instead of VOO found that all metabolites peaked at 60 min, offering a useful comparison with other oil-based matrices.

The development of highly sensitive analytical method (limit of detection 0.3 ng/mL) for quantifying free HT in plasma [23] showed that ingestion of 25 mL of VOO (≈1.38 mg of HT) yielded a Cmax of 4.4 ng/mL at approximately 15 min post-intake.

Phenol-enriched oils often yield higher total metabolite concentrations but may delay Tmax, possibly due to interactions between phenolics and the lipid matrix. For instance, a comparative pharmacokinetic study [15] between standard VOO and phenol-enriched oil (6× higher phenolic content) revealed a delayed Cmax (125 min vs. 60 min) and higher total metabolite concentrations with enriched oil. In contrast, another trial administering 5 mg HT with either EVOO or refined olive oil [18] found no significant differences in Cmax (both ~30 min), supporting the hypothesis that HT dose may be a stronger determinant of bioavailability than the oil type.

Once absorbed, HT undergoes extensive phase I (hydrolysis of secoiridoid) and phase II metabolism, yielding sulfate and glucuronide conjugates generated by sulfotransferases (SULTs) or UDP-glucuronosyltransferases (UGTs), respectively. The 3′-O-sulfate is typically the most abundant. Methylation via catechol-O-methyltransferase (COMT) produces homovanillyl alcohol (HvOH) and homovanillic acid (HVA), particularly after high doses [24]. Oxidative metabolism leads 3′,4′-dihydroxyphenylacetic acid (DOPAC) via the intermediate 3′,4′-dihydroxyphenylacetaldehyde (DOPAL), a highly reactive and cytotoxic compound [25], catalyzed by aldehyde dehydrogenase (ALDH). DOPAL can also arise endogenously from dopamine deamination (via monoamine oxidase, MAO) in the central nervous system (CNS), followed by aldehyde reductase (ALR) reduction to regenerate HT [11]. This endogenous pathway is particularly active in dopaminergic brain regions, suggesting potential central bioactivity. DOPAC retains antioxidant properties [26] and can be methylated to HVA or conjugated by SULFs/UGTs. Additional metabolites, such as HT-acetate-4′-sulfate [16] and mercapturates like *N*-acetyl-5-S-cysteinyl-HT, arising from HT autoxidation [27], have also been reported.

**Figure 1:**
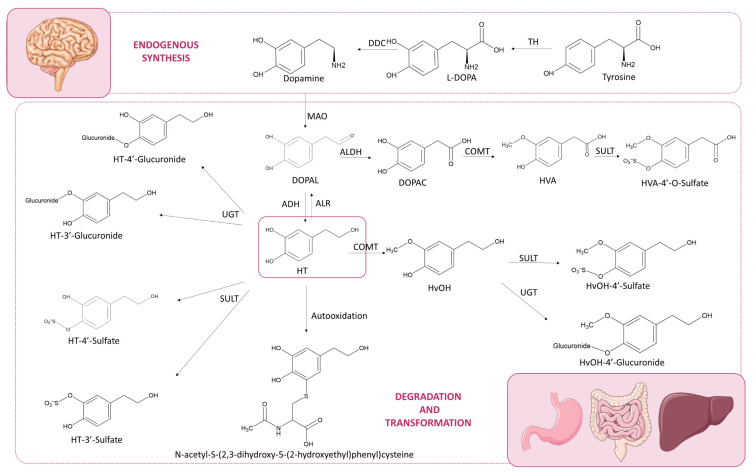
Metabolic Pathways of Hydroxytyrosol: Endogenous synthesis, Biotransformation, and Degradation. Enzymes involved: UDP-glucuronosyltransferases (UGTs), sulfotransferases (SULTs), catechol-*O*-methyltransferase (COMT), aldehyde dehydrogenase (ALDH), alcohol dehydrogenase (ADH), monoamine oxidase (MAO), aldehyde reductase (ALR),tyrosine hydroxylase (TH), DOPA decarboxylase (DDC). Main metabolites: hydroxytyrosol (HT), homovanillyl alcohol (HvOH), homovanillic acid (HVA), 3′,4′-dihydroxyphenylacetic acid (DOPAC), 3′,4′-dihydroxyphenylacetaldehyde (DOPAL), alcohol derivative of HVA (HvAlc), N-acetyl-5-S-cysteinyl-hydroxytyrosol.

These transformations (Figure 1) occur rapidly, with most HT metabolites cleared from plasma within ~4 h. While free HT is bioactive, some conjugates may retain or even enhance biological effects, though their roles remain less understood [28,29]. This highlights the importance of identifying predominant metabolites according to the consumption matrix, as this knowledge could guide the formulation of dietary supplements. To this end, some studies with well-designed approaches to explore HT biotransformation are detailed below.

A bioavailability study [24] specifically evaluated the role of COMT in HT metabolism, using four olive oils enriched with increasing HT concentrations. A clear dose-dependent relationship was observed between HT intake and urinary excretion of HvOH and HVA, with HVA being ~2× more abundant. In another study [21], ingestion of 25 mL VOO led to higher plasma concentrations for HT (25.83 µg/L) compared to HvOH (3.94 µg/L). For both, most excretion occurred within the first two hours post-ingestion. High interindividual variability was observed, with urinary HT levels ranging from 300 to 400 µg, and approximately 100 µg for 3-O-methyl-HT.

A subsequent study [30] compared single high-dose versus sustained intake of HT and tyrosol. A total dose of 1370 µg of HT and 1720 µg of tyrosol was administered either as a single 50 mL VOO dose or as daily 25 mL doses for one week. Urinary tyrosol excretion decreased whereas HT excretion increased by 1.5-fold after repeated intake. Notably, urinary HT after one day exceeded the ingested amount, suggesting endogenous contribution or bioconversion from other phenolic precursors.

Expanding the evidence on conjugated forms, urinary metabolites were evaluated in 11 volunteers after 50 mL of VOO (22 µM HT, 12 µM tyrosol) [31]. The most abundant urinary metabolite was tyrosol-4′-O-glucuronide (6.65 ± 2.91 µM), followed by HT-4′-O-glucuronide (3.89 ± 1.69 µM), HT-3′-O-glucuronide (2.32 ± 1.31 µM), and HvOH-4′-O-glucuronide (2.66 ± 1.18 µM). The majority of metabolites appeared within 6 h post-intake.

A follow-up study [32] validated a quantification method for glucuronidated metabolites after 50 mL VOO. About 10–13% of the ingested dose was recovered as glucuronides, with 4.5% as HvOH, mainly as 4′-O-glucuronide. Again, tyrosol-4′-O-glucuronide (1.49 µmol) was most abundant, followed by HT-4′-O-glucuronide (0.67 µmol), HT-3′-O-glucuronide (0.63 µmol), and HvOH-4′-O-glucuronide (0.63 µmol). Interindividual variability was a consistent finding.

Fortified oils added further insights. A randomized crossover trial in 13 participants compared standard and phenol-enriched EVOO [15]. After standard EVOO, plasma HT sulfate (0.53 ± 0.30 µmol/L) and HVA sulfate (0.78 ± 0.46 µmol/L) were detected. The enriched EVOO increased both (0.86 ± 0.24 µmol/L and 0.96 ± 0.88 µmol/L, respectively), and delayed Tmax to 65 min, consistent with earlier reports.

Another intervention tested three enriched VOOs (250, 500, 750 mg/kg total phenols) in 12 participants [33]. After 30 mL intake, plasma analysis revealed that HT sulfate was the predominant metabolite, with Cmax values of 1.35 ± 0.72 µmol/L, 3.32 ± 1.56 µmol/L, and 4.09 ± 1.99 µmol/L, respectively. HT acetate sulfate appeared at 0.46 ± 0.26 µmol/L, 1.89 ± 1.63 µmol/L, and 2.24 ± 0.51 µmol/L, while HVA and its sulfates were less abundant. Again, large interindividual differences were observed across all doses.

The matrix effect was further examined with 5 mg HT administered in EVOO or refined olive oil [18]. Only free HT was quantified in plasma, with concentrations of 3.7 ng/mL (EVOO) and 1.5 ng/mL (refined oil). Urinary HT levels were also higher with EVOO (0.86 μg/mg creatinine) than with refined oil (0.63 μg/mg creatinine), supporting EVOO as a more favorable matrix. Interpretation remains limited by the lack of comprehensive metabolite analysis in plasma; however, urinary measurements also detected HT-acetate, DOPAC, and HvOH, indicating broader metabolic conversion.

**Table 1:** Human studies on hydroxytyrosol bioavailability from virgin olive oil consumption.

Ref.	Overview	HT Source	Administered Dose	Plasma Presence			Urine		
				Metabolites	Peak Time	Maximum Concentration	Metabolites	Peak Time	Total Amounts/Concentrations
[21]	Bioavailability study after methodological development for plasma and urine sample analysis for quantification of HT and 3-O-methyl HT and the evaluation of extensive intestinal and hepatic metabolism	Virgin olive oil (VOO)	25 mL VOO	HT 3-*O*-methyl-HT	32 min (0.58 h) 53 min (0.88 h)	25.83 μg/L 3.94 ug/L	HT 3-*O*-methyl-HT	0–2 h	300–400 ug ** ~100 ug
[22]	Characterization of olive polyphenol absorption and metabolism, with detection of glucuronide conjugates and DOPAC, early peak concentrations in plasma, and enhancement of systemic antioxidant status (n = 8)	kalamon olives	100 g kalamon olives	HT HvOH HVA DOPAC	60 min 60 min 60 min 60 min	3.145 ± 0.341 mg/mL 0.122 ± 0.013 mg/mL 2.263 ± 0.170 mg/mL 14.742 ± 0.941 mg/mL	HT HvOH HVA DOPAC	0–4 h 0–4 h 0–4 h 0–4 h	4.762 ± 1.296 mg/h ** 9.116 ± 3.596 mg/h 0.025 ± 0.004 mg/h 17.866 ± 11.275 mg/h
[23]	Method development to detect free HT in extra virgin olive oil allowed to detect EVOO CMax peak at 15 min	Extra Virgin olive oil (EVOO)	1.38 mg HT (25 mL)	Not determined Only free HT	15 min	4.4 ng/mL			
[15]	Evaluation of the enrichment of EVOO through a bioavailability study. Results indicated higher levels of HT sulfate and vanillin sulfate	Fortified (EVOO) and VOO	VOO (Control) 30 mL Fortified EVOO: 30 mL (6× phenol content)	HT-S HVA derivative HVA-S HT-S HVA derivative HVA-S	60 min 125 min	0.53 ± 0.30 µmol/L 0.53 ± 0.29 µmol/L 0.78 ± 0.46 µmol/L 0.86 ± 0.24 µmol/L 0.34 ± 0.10 µmol/L 0.96 ± 0.88 µmol/L			
[18]	Demonstration of matrix-dependent bioavailability, with extra virgin olive oil identified as the optimal matrix Results are summarized in different corresponding tables. HT maximum concentration peak was detected at 30 min. (n = 20)	HT added to EVOO HT added to refined olive oil	EVOO 5 mg HT in 20 mg refined olive oil	HT HT	30 min 30 min	3.79 ng/mL 1.5 ng/mL	HT HT-Acetate DOPAC HvOH HT HT-Acetate DOPAC HvOH		0.86 μg/mg creatinine ~50 μg/mg creatinine ~40 μg/mg creatinine ~25 μg/mg creatinine 0.63 μg/mg creatinine ~100 μg/mg creatinine ~50 μg/mg creatinine ~20 μg/mg creatinine
[24]	Characterization of HT bioavailability, demonstrating COMT-mediated metabolism and dose-dependent formation of metabolites, as assessed by homovanillic alcohol and acid levels.	Four VOO samples accompanied by 40 g of bread	7 mg HT in 50 mL oil 13.3 mg HT in 50 mL oil 19.2 mg HT in 50 mL oil 23.2 mg HT in 50 mL oil				HT HvOH HVA HT HvOH HVA HT HvOH HVA HT HvOH HVA		267 μg (16.8%) * 367 μg (22%) 1027 μg (61.8%) 1328 μg (29.8%) 749 μg (16.8%) 2380 μg (53.4%) 1112 μg (21.8%) 1137 μg (22.3%) 2837 μg (55.8%) 1653 μg (23.7%) 1567 μg (22.4%) 3752 μg (53.9%)
[30]	Trial-based investigation (n = 7) of tyrosol and HT absorption after moderate and sustained virgin olive oil intake, and evaluation of their potential as intake biomarkers. Higher HT recovery was observed after sustained virgin olive oil intake compared to a single-dose administration	VOO	Single dose: 50 mL VOO (1370 µg HT) (1720 µg tyrosol) Sustained doses: 25 mL/day during a week (685 µg HT) (860 µg tyrosol)				Single dose: HT Sustained doses: HT		78.5 ± 14.1% 121 ± 45.9%
[31]	Intervention study (n = 11) carried out to identify the main conjugated metabolites derived from free phenols, with the aim of assessing their chemical and in vitro biological antioxidant activities at physiologically relevant concentrations. Glucuronide conjugates of HT and homovanillic alcohol (HvOH) were measured. However, these metabolites did not exhibit significant antioxidant activity under the tested concentrations (0.01–10 µM)	VOO	50 mL				T-4-G HvOH-4-G HT-4-G HT-3-G	0–6 h 0–6 h 0–6 h 0–6 h	6.65 ± 2.91 μM 2.66 ± 1.18 μM 3.89 ± 1.69 μM 2.32 ± 1.31 μM
[32]	Validation of a UPLC-MRM method for the simultaneous quantification of glucuronidated metabolites of olive oil phenols in human urine, showing that approximately 13% of the ingested dose was excreted within 24 h, mainly as glucuronidated forms	VOO	50 mL VOO with bread (22.0 ± 1.4 µmoles HT ≈ 3.38 ± 0.22 mg HT)				HT HT-4-G HT-3-G HvOH HvOH-4-G	0–6 h 0–6 h 0–6 h 0–6 h 0–6 h	0.39 µmol 0.67 µmol 0.95 µmol 0.31 µmol 0.63 µmol
[33]	A randomized cross-over study in 12 healthy volunteers evaluated the pharmacokinetics of phenolic metabolites from three phenol-enriched virgin olive oils revealing a dose-dependent increase in HT sulfate (main plasma metabolite) and identifying HT acetate sulfate as a key novel metabolite.	Three phenol enriched VOO 250, 500, 750 mg/kg total phenols	2.23 mg total oleuropein derivatives (30 mL oil) 6.27 mg total oleuropein derivatives (30 mL oil) 10.87 mg total oleuropein derivatives (30 mL oil)	HT-S HT- Ac-S HVA HVA-S HT-S HT-Ac-S HVA HVA-S HT-S HT- Ac-S HVA HVA-S	1 h 1 h 1.5 h 1 h 1 h 2 h 1 h 1 h 1.5 h 1 h 1 h 1 h	1.35 ± 0.72 µmol/L 0.46 ± 0.26 µmol/L 0.17 ± 0.19 µmol/L 0.12 ± 0.15 µmol/L 3.32 ± 1.56 µmol/L 1.89 ± 1.63 µmol/L 0.63 ± 0.30 µmol/L 0.27 ± 0.25 µmol/L 4.09 ± 1.99 µmol/L 2.24 ± 0.51 µmol/L 0.65 ± 0.41 µmol/L 0.53 ± 0.62 µmol/L			
[34]	Bioavailability study of fortified oil vs. other aqueous enriched extracts. HVA and DOPAC were the main metabolites found in urine followed by HT-3-S	HT-Fortified olive oil	25 mL oil				HT-3-S HT-4-S HT-3-G HT-4-G HVA DOPAC		2.57 ± 1.17 μmol * 0.05 ± 0.19 µmol 0.06 ± 0.09 µmol 0.01 ± 0.02 µmol 4.82 ± 3.93 µmol 4.53 ± 1.76 µmol
[35]	Evaluation of HT pharmacokinetics showed that an enteric coated EVOO based capsule significantly improved its bioavailability, highlighting the impact of formulation (n = 20)	HT enteric coated EVOOO based capsule	7.5 mg HT	HT	123 min	4.493 ± 0.106 ng/mL	Sulfo-conjugated derivatives of HT HVA Glucurono-conjugated derivatives DOPAC HT	6 h 6 h 6 h 6 h 6 h	19.46 µmol 18.39 μmol 11.48 μmol 9.93 μmol 4.67 μmol

* Only total amounts were determined. ** Amounts are obtained from the major excretion fraction.

Another trial on 25 mL HT-enriched olive oil in 12 volunteers [34] reported HVA (4.82 ± 3.93 μmol) and DOPAC (4.53 ± 1.76 μmol) as the most abundant urinary metabolites, followed by HT-3-sulphate (2.57 ± 1.17 μmol). Glucuronides and 4-sulfated conjugates were lower, reinforcing the predominance of 3-*O*-sulfation.

Alternative delivery systems, such as enteric-coated capsules containing 7.5 mg HT in an EVOO base, confirmed sulfation as the main conjugation pathway [35], with 19.46 μmol of sulfates detected, followed by 18.39 μmol HVA, 11.48 μmol glucuronides and 9.93 μmol DOPAC.

Preclinical models complement human findings, showing rapid systemic distribution of HT to liver, kidneys, brain, and red blood cells. Radiolabeled HT appears in tissues within 5 min, with the liver and kidneys as major accumulation sites. Even at nutritionally relevant doses (1, 10, and 100 mg/kg body weight) [36], HT preferentially accumulates in liver and kidneys, supporting its potential role in hepatic and renal health.

### 3.2. Hydroxytyrosol Bioavailability from Supplements and Non-Olive Oil Matrixes

Data from studies on olive oil consumption provide an important reference for understanding how HT behaves under physiological conditions. This information is particularly valuable when considering the design and evaluation of HT as a dietary supplement. Although purified HT can be efficiently absorbed, its pharmacokinetic profile may differ significantly from that observed when consumed within the olive oil matrix, mainly due to the matrix effects and interactions with other phenolics present in EVOO. Therefore, translating the health benefits associated with olive oil consumption into supplement form requires careful consideration of these differences. Further studies are needed to establish whether isolated HT can reproduce the biological effects of regular olive oil intake.

A recent review [37] emphasized that the cytotoxic and cancer-preventive effects of HT observed in vitro and in animal models are achieved at concentrations far exceeding those attainable through diet or oral supplementation in humans. This highlights the urgent need for well-designed human bioavailability studies to determine whether effective systemic concentrations can realistically be reached through dietary intake or supplementation, or alternatively, to replicate preclinical studies in humans to confirm the reported bioactivity. Human bioavailability studies of HT conducted after the administration of dietary supplements (Table 2) or enriched foods (Table 3) are detailed below.

Human studies with isolated HT are scarce, and because of its rapid metabolic conversion, unmetabolized HT is rarely detected in blood or urine. Thus, metabolite profiling is essential to assess systemic exposure. One of the earliest studies [38] administered 2.5 mg HT/kg body weight in aqueous solution. Free HT and HvOH were detected in plasma, peaking at 13 ±1.5 min and 16.7 ± 2.4 min, respectively, with maximum concentrations of 1.11 ± 0.20 μmol/L for HT and 0.49 ± 0.14 μmol/L for HvOH. Both declined sharply after 10 min, becoming undetectable after ~1 h, with half-lives of 8.6 ± 0.7 min and 8.65 ± 1.76 min for HT and HvOH, respectively.

Absorption was also studied in 8 healthy subjects and 12 ileostomy patients given different supplements containing 100 mg of olive oil phenols [39]. After the intake of an aqueous supplement containing polar phenols, HT was minimally detected in ileostomy effluent (<4 mol/100 mol of intake), indicating efficient small intestine absorption. Urinary recovery was 5–6% of the administered dose, confirming systemic bioavailability from polar supplements in both groups.

A comparative assessment tested olive leaf extract in capsules versus liquid across various doses [17]. Liquid delivery produced a higher and earlier plasma peak (53 ± 29 min) for HT-sulfate and HVA-sulfate compared to capsules. These findings contrast with earlier evidence of prolonged systemic exposure after enteric-coated EVOO capsules [35], where HT reached a Cmax of 4.49 ± 0.11 ng/mL with a Tmax of 123 min, accompanied by sulfate- and glucuronide-conjugates, HVA and DOPAC, confirming prolonged systemic exposure. Formulation could explain this discrepancy since enteric coating delays release sustains exposure, while liquids favor rapid absorption. An optimal design should balance peak concentration with duration of action, making enteric-coated delivery systems preferable when sustained exposure is desired.

Urinary metabolite profiles after supplementation with Hytolive^®^ (10% natural HT extract) were evaluated in 11 healthy volunteers receiving 5 mg or 25 mg doses [40]. Free HT was absent in urine, while glucuronidated and sulfated conjugates were prominent. For 5 mg dose, urinary excretion included 0.11 mg HT-4-glucuronide, 0.14 mg HT-3-glucuronide, 1.18 mg HT-3-sulfate, and 0.07 mg HT-4-sulfate. With 25 mg dose, values increased to 0.46 mg, 0.72 mg, 4.15 mg, and 0.07 mg, respectively. HT-3-sulfate was predominant, accounting for ~16.6% (low dose) and 23.1% (high dose) of intake.

Another trial [34] tested an olive-derived water-based supplement (30.58 mg and 61.48 mg HT). Plasma metabolites peaked at approximately 30 min. For the lower dose, levels were 384.96 ± 62.63 nmol/L HT-3-sulfate, 868.86 ± 111.86 nmol/L HVA, and 301.07 ± 13.43 nmol/L DOPAC. The higher dose produced only moderate increases (406.28 ± 32.68 nmol/L, 948.76 ± 160.19 nmol/L and 466 ± 147.68 nmol/L, respectively), suggesting possible saturation. Total urinary excretion identified DOPAC as the predominant metabolite (44.1 µmol), followed by HVA (36.1 µmol) and HT-3-S (16.6 µmol) at low dose, compared with 59.7, 46.2 and 20.05 µmol, respectively, at the high dose. Their excretion did not scale with dose, indicating metabolic regulation at high intake.

Fortified foods represent another delivery approach (Table 3). In one comprehensive study [41], HT-enriched biscuits (5.25 mg HT/30 g) were consumed. Eight metabolites were detected in plasma, mainly sulfated HT (1.0 ± 0.03 µmol/L) and DOPAC (0.5 ± 0.1 µmol/L), peaking at 0.5–1 h. In contrast to isolated supplements, glucuronides were also present, though at lower levels than sulfates, underscoring the matrix effect. Most urinary metabolites were excreted within 0–3 h, except DOPAC-glucuronide, which peaked between 12 and 24 h.

Other non-olive oil matrices have been tested. In a study [18], 5 mg HT was added to flaxseed oil, grapeseed oil, margarine, and pineapple juice (20 g portions). No free HT was detected in plasma for these matrices, whereas olive oil delivery produced detectable plasma levels (Cmax ~3.79 ng/mL at 30 min in EVOO and ~1.5 ng/mL at 30 min in refined oil). Urinary excretion was highest for EVOO (0.86 µg/mg creatinine for HT, plus ~50, ~40 and ~25 µg/mg for HT-acetate, DOPAC and HvOH, respectively), followed by fortified refined olive oil (0.63 µg/mg creatinine for HT, plus ~100, ~50 and ~20 µg/mg for HT-acetate, DOPAC and HvOH, respectively), while other matrices yielded ~0.3 µg/mg creatine for free HT, accompanied by variable amounts of HT-acetate, DOPAC and HvOH (ranging from ~5 to 40 µg/mg depending on the matrix). Only olive oil matrices significantly increased the formation of these metabolites, confirming that the food matrix, particularly lipid-rich ones such as olive oil, strongly modulates HT bioavailability.

In summary, HT from supplements or alternative matrices is absorbed, but its bioavailability is strongly influenced by formulation, dose, and matrix composition. Sulfate and glucuronide conjugates predominate in plasma and urine, and absorption kinetics are modulated by factors such as fat content and delivery system. These characteristics make it challenging to replicate benefits of olive oil through supplements and highlight the need for further human studies to optimize formulations. Absorption is closely linked to digestion, metabolism, and delivery processes, which should be considered when interpreting results. In vivo studies cannot discriminate the main metabolites generated in situ due to hepatic portal circulation; therefore, the most reliable way to study metabolic pathway of HT is by using labeled molecules [25,42]. In addition, most human studies are limited by small sample sizes, high interindividual variability, and differences in analytical sensitivity, all of which may influence the interpretation and comparability of results. These methodological constraints should also be considered alongside safety aspects. Regarding safety, EFSA has concluded that HT is safe as a novel food at intakes up to 250 mg/day in adults, with no genotoxic or subchronic toxicity reported. Cytotoxic effects have only been observed in vitro at supraphysiological concentrations, which are not relevant to dietary or supplemental exposures [8]. It is also important to account for metabolites produced by the intestinal microbiota, which will be reviewed later.

### 3.3. Approaches to Modulate Bioavailability, Stability and Antioxidant Capacity of Hydroxytyrosol

The interest in HT within the agri-food sector is unquestionable. However, its application in the formulation of functional foods or dietary supplements requires a thorough evaluation of the factors that may affect its bioavailability and, consequently, its bioactivity. Antioxidant potency is undoubtedly one of the most relevant properties for HT use in the food industry, not only because it can influence the quality of fortified food products, but also due to its potential health benefits for consumers. Nevertheless, the hydroxyl groups of HT are highly sensitive to external factors such as light and oxygen [43], making the development of protective delivery systems essential to preserve its antioxidant capacity.

One particularly interesting application is in lipid-based products, which often require antioxidants to prevent autoxidation. The availability of lipophilic antioxidants is limited, and the search for alternatives remains a pressing need. HT is a polar molecule, and this property restricts its direct use in fat-rich matrices. However, numerous delivery strategies have been proposed to overcome this limitation and enable its incorporation into fats and oils.

**Figure 2:**
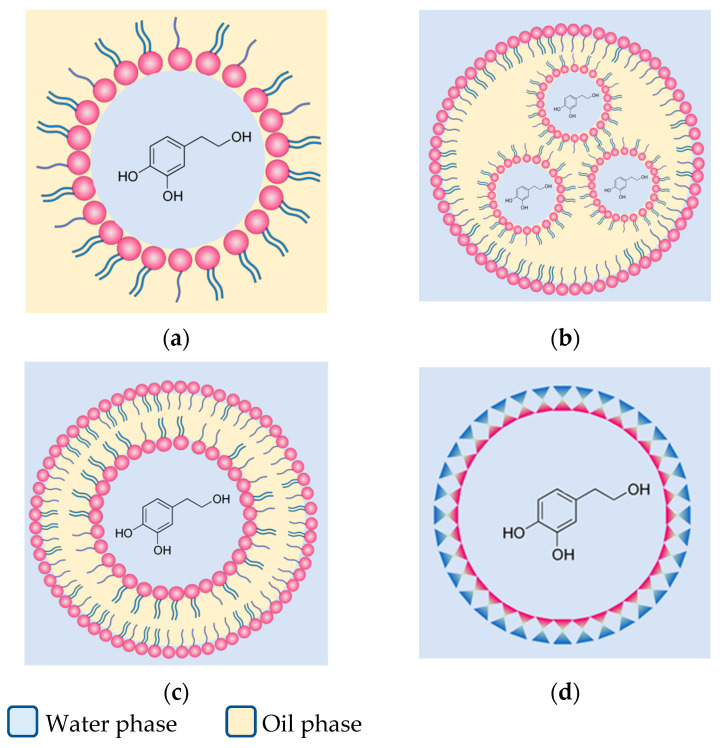
Delivery systems for hydroxytyrosol encapsulation. Schematic representation of (**a**) water-in-oil (W/O) emulsion, (**b**) water-in-oil-in-water (W/O/W) double emulsion, (**c**) liposome, and (**d**) polymer-based encapsulation system.

Considering the bioavailability, stability, and antioxidant capacity of HT, delivery systems based on colloids, such as emulsion-based systems (macroemulsions, microemulsions, double emulsions, gelled double emulsions), encapsulated particle systems (micro/nanospheres, micro/nanocapsules), vesicular systems (liposomes), and packaging systems (active packaging), offer promising solutions for HT incorporation into foods (Figure 2) [44].

#### 3.3.1. Emulsions

Emulsions are among the most widely explored delivery systems for HT, particularly due to their ability to enhance stability and modulate release profiles. These systems are stable mixtures of two immiscible liquids (e.g., water and oil), stabilized by amphipathic molecules such as surfactants or emulsifiers. Two main types can be distinguished: (1) water-in-oil (W/O) emulsions, where polar droplets are dispersed in oil, and (2) oil-in-water (O/W) emulsions, where oil droplets are dispersed in water. The molecular distribution of HT within these systems is governed by two partition constants: PIW (interface-water equilibrium) and PIO (interface-oil equilibrium). For polar molecules like HT, PIW is the primary determinant [45].

Emulsions are generally classified as conventional or macroemulsions (droplet size 100 nm–100 µm, thermodynamically unstable due to high surface tension) or microemulsions (5–50 nm, thermodynamically stable due to low surface tension). HT tends to localize at the droplet interface in W/O emulsions, raising questions about which type of emulsion might be more suitable. Comparative studies have evaluated the physical structure and antioxidant activity of conventional and microemulsions containing HT [46], reporting similar efficiencies. However, microemulsions exhibited superior stability, whereas conventional emulsions facilitated a higher release rate. Complementary research has examined O/W emulsions for HT delivery [45]. One study assessed the distribution of HT and an its acylated derivative using kinetic models, demonstrating that the addition of Tween 80 markedly increased HT interfacial localization, from over 40% at a surfactant volume fraction of 0.005 to over 80% at 0.04, and that hydroxytyrosyl acetate exhibited even higher interfacial presence and antioxidant efficiency.

Another promising method is the double emulsion system (W/O/W)**,** in which water droplets containing HT are encapsulated within oil droplets, which are in turn dispersed in an external aqueous phase. This multilayer arrangement enables the encapsulation of hydrophilic compounds within lipid phases, offering protection against oxidation and controlled release in food matrices.

Gelled double emulsions are an evolution of this system, where the double emulsion is embedded within a hydrogel matrix made from high molecular weight gums (e.g., alginate, pectin, gellan gum). This gel network improves physical stability, reduces phase separation, and can help limit compound losses during storage or digestion, while also modulating the release of HT.

Comparative studies have provided valuable insights into the performance of simple, double and gelled double emulsions for HT delivery. It has been reported that [47], double emulsion showed a very high initial HT recovery (~99.5%) after preparation, indicating minimal losses at this stage. The most substantial declines were observed during the gelation step to obtain gelled double emulsions and throughout storage, with losses increasing in more compartmentalized systems, likely due to their larger interfacial area. This interpretation is supported by a review [48], which notes that emulsions with higher compartmentalization and surface area tend to experience faster antioxidant depletion, although the review does not specify whether this occurs during preparation or storage. From a functional perspective [49], it was demonstrated that gelled double emulsions offered the greatest protection of HT during in vitro digestion, with bioaccessibility values of 89% compared to 79% in simple emulsion and double emulsion, and also slowed triacylglycerol hydrolysis (36.4% in gelled double emulsions vs. 22.7% in simple emulsion and 24.8% in double emulsion). These findings highlight that while compartmentalization may compromise storage stability, it can enhance protection and modulate release during digestion.

Finally, organogelled emulsions, in which HT and its esters (HT decanoate and octanoate) are immobilized within an oleogel matrix, have shown improved oxidative stability, preserved antioxidant activity, and high bioaccessibility (~84%), positioning them as another promising and technologically versatile strategy for HT delivery. Finally, and in another work [50], organogelled emulsions, originally designed as fat replacers, in which HT and its esters (HT decanoate and octanoate) are immobilized within an oleogel matrix, have demonstrated mechanical properties comparable to lard, improved oxidative stability, preserved antioxidant activity, and high bioaccessibility (~84%) for compounds of varying hydrophobicity, positioning them as a promising and technologically versatile strategy for HT delivery.

#### 3.3.2. Liposomes

Liposomes have emerged as an innovative delivery system for HT, particularly when aiming to enhance its stability and control its release in biological environments. Liposomes are nanoscale vesicles (15–1000 nm in size) composed of one or more phospholipid bilayers that encapsulate an internal aqueous phase. Their amphiphilic structure allows them to entrap both hydrophilic and lipophilic compounds, offering protection against environmental stressors such as oxidation, pH variations, and enzymatic degradation. In the case of HT, liposomes not only safeguard its sensitive hydroxyl groups from oxidative degradation but also enable a controlled and sustained release, which could be beneficial for maintaining plasma levels over time. Furthermore, the integration of HT into lipid bilayers may enhance its interaction with biological membranes, improving cellular uptake.

The antioxidant efficacy of HT-loaded liposomes compared to vesicles incorporated into oil-in-water emulsions was evaluated [51]. The results demonstrated that liposomes exhibited superior antioxidant activity and structural stability, as assessed by conjugated autoxidizable triene assays. The study highlighted that vesicles tend to lose efficacy due to physical instability, such as aggregation or fusion, which can compromise the retention and release of encapsulated HT. In contrast, liposomes maintained their integrity, ensuring a more efficient antioxidant activity.

Despite these advantages, conventional liposomes present limitations regarding their stability in the gastrointestinal tract, particularly due to extreme pH conditions, bile salts and digestive enzymes [52]. These factors can induce micellization and degradation of liposomes, leading to the premature release of their contents and reducing their effectiveness as oral delivery systems. This instability is specifically problematic when encapsulating labile molecules such as HT, which is prone to oxidative degradation once exposed.

To address this issue, polymer-coated liposomes have been proposed to enhance stability and protect their cargo throughout digestion. Surface modification with biocompatible polymers such as polyethylene glycol (PEG) or pH-responsive materials like Eudragit^®^ S100 provides a physical barrier that shields liposomes from degradation while modulating their release profile. This approach was investigated by evaluating three types of liposomal formulations for the oral co-delivery of curcumin and HT under simulated gastrointestinal conditions [53]: (1) conventional (uncoated) liposome, (2) PEG-2000-coated liposomes and (3) dual-coated liposomes with PEG-2000 and Eudragit^®^ S100. Their findings demonstrated that dual-coated liposomes significantly improved HT retention, reducing losses to 35% compared with 55% in conventional formulations. Eudragit^®^ S100 provided protection during gastric digestion by preventing early release at acidic pH, while PEG enhanced resistance against bile salts and digestive enzymes in the intestinal phase. This dual-layer system thus offers a controlled and site-specific release of HT, making it a promising strategy for oral nutraceutical applications.

Despite these advances, it is important to highlight that to date no human studies have evaluated the bioavailability or bioaccessibility of HT delivered via polymer-coated liposomes. Further clinical research is needed to validate the efficacy of these systems in vivo and to determine their potential for improving systemic exposure to HT.

#### 3.3.3. Encapsulation Systems

Apart from liposomes, polymeric encapsulation systems offer an alternative approach to enhance the stability and bioavailability of HT. These technologies involve entrapping HT within a protective polymeric structure, either by encasing it in a hollow cavity enclosed by a polymeric shell (capsules) or by embedding it within a solid polymer matrix (spheres). The latter includes micro- and nanoparticles, typically ranging from 1 to 1000 μm and 10–1000 nm, respectively [44]. The choice of encapsulating material is critical and may include natural polymers such as chitosan, gelatin or albumin; lipid-based carriers like triacylglycerols or waxes, or synthetic polymers such as polylactide (PLA) and polylactide-co-glycolide (PLGA). These systems offer the dual advantage of protecting HT from degradation caused by environmental factors (light, oxygen, pH fluctuations, enzymatic reactions) and allowing for controlled release, through mechanisms such as surface dissolution, diffusion through the polymer matrix, or erosion of the encapsulating material [54].

Although relatively few studies have investigated HT encapsulation systems, the existing research suggests a high potential for targeted delivery applications. A glycyrrhetinic acid (GA)-functionalized nanoparticle system was developed to co-delivery syringopicroside and HT, aiming for liver-specific accumulation [55]. Using polyethylene glycol-PLGA polymers conjugated with GA, nanoparticles were produced with a size of approximately 100 nm, achieving an encapsulation efficiency of around 50% and a drug loading capacity of 15.5%. Pharmacokinetic studies in rodents revealed that encapsulated HT preferentially accumulated in liver tissue compared to free HT or non-functionalized nanoparticles, highlighting the potential of ligand-targeted nanocarriers for organ-specific delivery.

In another approach, phenylboronic acid-grafted chitosan nanocapsules were developed to co-encapsulate VOO and HT [56]. This system aimed to achieve a controlled and sustained release profile. The encapsulation efficiencies were notably high (97.8% for VOO and 81.3% for HT); however, the formulation process required the use of excipients such as Tween 80 for VOO and DMSO for HT, which could pose challenges for food-grade applications. Nonetheless, these nanocapsules demonstrated remarkable functional properties, including enhanced antioxidant activity (evaluated via DPPH radical scavenging and lipid peroxidation inhibition), as well as antimicrobial and anticancer effects in vitro, while maintaining low cytotoxicity toward normal cells. Importantly, the nanocapsules exhibited a gradual and controlled release of both VOO and HT, which is essential for maintaining bioactive concentrations in target tissues. Zeta potential measurements and particle size distribution analyses further confirmed the colloidal stability of the system, supporting its potential application in nutraceutical formulations that require extended bioactivity and targeted release.

These findings underscore the potential of nanoencapsulation to significantly improve functional performance of HT, particularly in terms of stability, controlled release, and bioactivity. However, the absence of human clinical studies evaluating the bioavailability of HT delivered through encapsulation systems remains a critical gap that needs to be addressed in future research. Figure 2 summarizes the principal strategies for hydroxytyrosol encapsulation, highlighting emulsion-based systems, liposomes, and polymer-based carriers.

#### 3.3.4. Chemical Modification: Phenolipids

Chemical modification of HT has been proposed as a promising strategy to preserve or even enhance its antioxidant properties, while improving its functionality in lipid environments. One widely explored approach is the esterification of HT with fatty acids to produce phenolipids, hybrid molecules with a polar phenolic head and a hydrophobic lipid tail. In these amphiphilic structures, the acyl chain associates with the oil phase while the phenolic group locates at the oil, water interface, acting as a barrier against lipid oxidation in emulsified or micellar systems. This structural modification may influence antioxidant efficiency according to the Antioxidant Polar Paradox Hypothesis [57], which states that hydrophilic antioxidants like HT are generally more effective in bulk oils, whereas lipophilic antioxidants perform better in systems with a high surface-to-volume ratio. By increasing lipophilicity, phenolipids could optimize interfacial positioning and potentially improve absorption by modulating polarity, although in vivo evidence remains scarce.

High-yield synthesis of both fatty acid esters [45,58,59,60,61,62] and alkyl ethers of HT [63,64] has been reported, producing derivatives with antioxidant capacity comparable to or greater than that of HT [45,58,60,61,62,65].

In vitro studies indicate that these lipophilic derivatives are bioavailable. For example, transport assays in differentiated Caco-2/TC7 monolayers showed that HT acetate crosses the epithelial barrier more efficiently than free HT, followed by hydrolysis to release the parent compound [66]. The influence of acyl chain length and ester bond structure has also been studied using digestive simulation models, showing that hydrolysis and transport rates rise with chain length up to an optimal point, then decline for the longest chains. This was attributed to a balance between greater membrane interaction and self-association that hindered permeability. Branched esters performed worse than straight-chain analogs, and rapid lipase hydrolysis in some cases could extend systemic availability through gradual HT release [67]. Regarding hydroxytyrosyl ethers, alkyl chains of different lengths (methyl, ethyl, propyl, butyl) exhibited rapid transepithelial transport (Papp = 32.6–43.5 × 10^−6^ cm/s) and partial metabolism, with rates increasing with lipophilicity (butyl > propyl > ethyl > methyl) [68].

Beyond absorption studies, ester and ether derivatives of HT have shown the ability to enhance cellular antioxidant status in vitro [69] and to exert cardiometabolic benefits in vivo. In vivo, chronic supplementation in hypercholesterolemic rats with HT, hydroxytyrosyl acetate (HT-Ac) or ethyl hydroxytyrosyl ether (HT-Et) improved glucose, insulin, leptin, oxidative stress markers and inflammation compared with cholesterol-fed controls, with HT-Ac showing the greatest efficacy [70].

A notable example of phenolipid design is the synthesis of HT bis-ester derivatives inspired by xanthophyll structure, linking two HT moieties via alkyl spacers of different lengths (C12, C16, C22) [71]. Results from this study show that these derivatives enhanced the oxidative stability of liposomes, with the C12 spacer being the most effective. Incorporation during liposome preparation further improved protection, highlighting that both chemical structure and method of integration influence antioxidant efficacy.

Despite these promising in vitro results, there is a clear lack of in vivo studies addressing the pharmacokinetics and bioefficacy of these phenolipids. Further research is needed to determine how these structural modifications affect absorption, metabolism, and biological activity in humans.

### 3.4. Gut Microbiota and Other Interindividual Factors Influencing Hydroxytyrosol Bioavailability

The human gut microbiota is a complex, dynamic and highly individualized ecosystem shaped by dietary habits, environmental exposure, and host genetic background [72,73]. It plays a central role in the biotransformation of dietary polyphenols such as HT, influencing both their pharmacokinetics and pharmacodynamics through the generation of bioactive metabolites. These metabolites can exert systemic effects, modulating immunity, energy homeostasis, and intestinal barrier integrity [74,75]. Given the marked interindividual variability in microbial composition and metabolic output, the concept of distinct “metabotypes” has emerged, defined by the specific metabolic signatures produced by individual microbiotas [76]. For phenolics like HT, identifying the microbial taxa, enzymes, and metabolic pathways responsible for these conversions is essential to understand their health effects and interindividual differences in bioavailability [77,78].

Emerging evidence positions the gut microbiota as a key determinant of the metabolic fate and bio-efficacy of HT. Early insights from in vitro studies have been confirmed in vivo, with the oxidation of HT into phenylacetic acids identified as the initial step of its microbial metabolism in a controlled human intervention, where ten healthy volunteers consumed 25 mL/day of phenol-rich olive oil for three weeks [79]. Further, in a rat model of chronic unpredictable mild stress, it was shown that glycosylated HT precursors, such as those in *Cistanche tubulosa*, require microbial hydrolysis to release bioactive HT and exert systemic effects, an activation step bypassed by free HT, thus limiting its local interaction with gut microbes [80]. Beyond small-intestinal metabolism, additional transformations occur in the colon via enterohepatic recirculation. Although HT is rapidly absorbed, it undergoes extensive hepatic glucuronidation and sulfation, and these conjugates may be deconjugated in the colon by microbial β-glucuronidases. This has been evidenced by the detection of hydroxyphenylpropionic acid sulfate (HPPAc-Sulf), hydroxyphenylacetic acid sulfate (HPAAc-Sulf), and hydroxyphenylacetic acid glucuronide (HPAAc-Glu) in the small intestine and caecum of Wistar rats after oleuropein ingestion, reflecting the recirculation of microbiota-derived metabolites via bile [81]. Such colonic transformations may be particularly relevant where low systemic bioavailability allows for enhanced local effects [82].

Microbial-derived metabolites such as 3,4-dihydroxyphenylacetic acid and hippuric acid in urine have been detected in urine after HT supplementation (15 mg/day), even in a single-subject proof-of-concept study [83], supporting the involvement of the gut microbiota in HT metabolism. This was further confirmed in a controlled trial in mildly hypercholesterolemic adults, where eight-weeks of olive pomace-enriched biscuit consumption significantly increased urinary HVA and DOPAC levels [84]. The phenolic composition of the delivery matrix also influenced metabolic outcomes. In a study with six healthy volunteers consuming three EVOOs differing in secoiridoid content, urinary profiles and amounts of microbial-derived products such as HVA and phenylacetic acids varied accordingly, suggesting that both absorption and microbial conversion are modulated by oil composition [85]. Additional human bioavailability studies have reported extensive phase II conjugation of HT, alongside urinary excretion of microbial metabolites (e.g., DOPAC, γ-valerolactones) consistent with colonic biotransformation and reabsorption. This colonic metabolism is further supported by in vitro fermentation of HT using human fecal inocula, where HT persisted for up to 24 h and was extensively converted into phenolic acids and other derivatives [86]

In addition to direct metabolic interactions, recent evidence indicate that gut microbes can indirectly modulate HT bioavailability by preserving epithelial barrier function. Microbial metabolites derived from HT, such as tauroursodeoxycholic acid (TUDCA), have been shown to enhance intestinal integrity in a porcine model of intestinal oxidative stress [87], placing this effect within the bile acid-microbiota axis, a bidirectional network linking microbial composition, bile acids, and host metabolism [88]. Complementarily, tyrosol-derived colonic metabolites have been reported to upregulate tight junction proteins and reduce paracellular permeability in Caco-2 monolayers [89]. Building on this bidirectional interaction, HT and tyrosol esters with short-chain fatty acids (SCFAs) have been synthesized to achieve targeted intestinal release and microbial hydrolysis by fecal bacteria and *Lactobacillus*, as demonstrated in in vitro gastrointestinal digestion and microbial fermentation models [90]. These findings highlight that structural modification of HT can influence not only its bioavailability but also crosstalk with the gut microbiota and its capacity to support intestinal barrier function.

Beyond microbial variability, host-related factors such as genetic polymorphisms, age, sex, and ethanol consumption also influence HT pharmacokinetics, as supported by multiple human studies. Sex-related differences have been observed in both phase II metabolism and microbial transformation, where women tend to reach higher plasma concentrations of HT glucuronides after the same dose [82], whereas men excrete greater amounts of microbial- and phase I-derived metabolites such as 3,5-dihydroxybenzoic acid, 4-hydroxyphenylacetic acid, and naringenin [84]. Preliminary evidence further suggests that males may conjugate oleuropein more efficiently, leading to higher AUC values for HT metabolites [17], though these findings require confirmation in larger, well-controlled cohorts. Age-related effects are less defined. In a gastrointestinal simulation model, younger individuals showed reduced microbial biotransformation of olive polyphenols compared with older adults, and effect attributed to lower viable bacterial counts rather than intrinsic physiological differences; both age groups produced the same metabolites, suggesting preserved microbial functionality with aging [91].

Overall, these findings emphasize the need for targeted in vivo studies and integrated analyses of host physiology and gut microbial ecology to better characterize interindividual metabotypes and their impact on HT metabolism.

## 4. Conclusions

The only EFSA-authorized health claim for HT refers to its consumption within olive oil, specifically for the protection of LDL particles from oxidative damage [6]. This claim cannot be directly applied to functional foods, supplements, or other products containing HT unless their efficacy has been independently demonstrated. It is therefore essential to verify the bioactivity of each formulation. Given the relationship between bioactivity and bioavailability, this review compiles and evaluates the current evidence on HT absorption and metabolism when administered in matrices other than EVOO.

Human studies consistently show that HT undergoes rapid metabolism, with sulfate and glucuronide conjugates, as well as methylated and oxidized derivatives, dominating the systemic profile. The food matrix significantly influences both the levels and the proportion of these metabolites, ultimately shaping potential health effects of HT. Free HT is rarely detected in plasma or urine, suggesting that its biological effects are largely mediated by its predominant metabolites. Among these, HT-3-S, produced through sulfotransferase activity, appears to be the most prevalent and stable metabolite in both plasma and urine, and could therefore serve as a potential biomarker of HT intake. Other consistently detected metabolites include HVA and DOPAC, even at low HT doses, highlighting methylation as a major metabolic pathway. Glucuronide conjugates are generally found at higher concentrations when HT is consumed within oily matrices such as EVOO. While EVOO remains the most studied and effective natural carrier, alternative delivery systems, including aqueous supplements, emulsions, capsules, and fortified foods, have demonstrated promising results, albeit with variable metabolic outcomes. These differences underscore the need for formulation-specific bioavailability studies rather than extrapolation from EVOO-based data.

Technological strategies to enhance HT stability and positively influence its bioavailability range from optimizing the carrier matrix to applying advanced delivery systems and chemical modifications. Among chemical approaches, phenolipids, particularly esters and ethers derived from HT, offer potential advantages by increasing lipophilicity, improving compatibility with lipid-based systems, and enabling synergistic formulations with bioactive lipids such as omega-3 fatty acids, among others. Encapsulation strategies (including polymeric micro/nanoparticles), liposomes, and emulsion-based systems (simple, double, gelled double, and organogelled), as well as chemical modification into phenolipids, can further preserve the antioxidant activity of HT, protect it from degradation, and modulate its release and bioavailability. However, their application in nutraceuticals is limited by scalability, cost, regulatory uncertainty, and consumer acceptance. In the case of olive polyphenols [92], showed that the market acceptance of EVOO enriched with higher polyphenol content obtained through ultrasound extraction depends on consumer segmentation, with innovation-oriented consumers more willing to adopt such products.

From a clinical perspective, the number of intervention studies with HT as a supplement, outside its natural matrix olive oil, remains very limited. Most trials have been performed with small sample sizes and short durations, but preliminary evidence points to improvements in lipid profile, oxidative stress markers, and inflammation. Nevertheless, larger and longer-term clinical studies are still required before drawing firm conclusions on the clinical applications of HT.

Overall, this review provides a comprehensive and up-to-date synthesis of HT bioavailability and delivery strategies. Its main limitations reflect the scarcity of long-term clinical evidence, methodological heterogeneity and the limited data on consumer acceptance.

Future progress in HT nutraceutical development will require the following: (i) well-designed clinical trials testing isolated HT, phenolipids, and other derivatives in diverse matrices; (ii) comprehensive metabolite profiling to link systemic exposure with health outcomes; (iii) formulation strategies that are technologically viable, legally compliant, and aligned with consumer preferences; and (iv) integration of personalized approaches that account for gut microbiota and inter-individual variability. The gut microbiota, as a key modulator of HT metabolism and bioavailability, should be considered integrative studies addressing host–microbe interactions. Only by addressing these scientific, technological, regulatory, and consumer-related aspects will it be possible to expand the use of HT beyond olive oil while ensuring both efficacy and safety.

## Figures and Tables

**Table 2:** Human studies on hydroxytyrosol bioavailability from dietary supplement intake.

Ref.	Overview	HT Source	Administered Dose	Plasma Presence			Urine		
				Metabolites	Peak Time	Maximum Concentration	Metabolites	Peak Time	Total Amounts/Concentrations
[38]	HT bioavailability study and lipoprotein binding dynamics assessment, following aqueous supplementation, showing rapid plasma appearance, excretion of metabolites (HVA, DOPAC-G), transient LDL association and absence of measurable antioxidant activity (n = 10)	Pure HT (99.5%) as supplement in aqueous solution	2.5 mg of HT per kg of bodyweight	HT HvOH	13.0 ± 1.5 min (10–20) min 16.7 ± 2.4 min (10–30) min	1.11 ± 0.20 µmol/L (0.47–2.23) µmol/L 0.49 ± 0.14 µmol/L (0.00–1.48) µmol/L	HT HT-G HT-S HvOH HvOH-G HvOH-S HVA HVAG HVA S DOPAC DOPAC-G DOPAC-S		0.1 ± 0.04 (0.00–0.35) µM (0.1%) * 0.91 ± 0.45 (0.00–4.92) µM (1.2%) 2.95 ± 1.93 (0.00–19.89) µM (3.8%) 0.00 ± 0.00 (000–0.01) µM (0.0%) 0.29 ± 0.19 (0.00–1.97) µM (0.4%) 0.20 ± 0.17 (0.00–1.98) µM (0.2%) 24.29 ± 14.98 (0.00–163.25) µM (31%) 7.93 ± 3.48 (0.00 ± 32.43) µM (10.1%) 9.08 ± 5.54 (0.00–55.90) µM (11.6%) 10.35 ± 5.63 (0.00–55.82) µM (13.2%) 17.76 ± 6.87 (0.00 ± 63.00) µM (22.7%) 4.41 ± 3.33 (0.00–36.41) (5.6%)
[39]	In a controlled crossover trial involving 8 ileostomy patients and 12 healthy volunteers, between 55 and 66% of ingested olive oil phenols were absorbed primarily in the small intestine, underwent metabolic transformation, and only 5–6% was recovered in urine	Watery fluid rich in polar phenols	Ileostomy volunteers: 498 µmol Healthy volunteers: 526 µmol	HT Tyrosol		Ileostomy Effluent: ** 1.8 µmol 1.4 µmol	HT Tyrosol HT Tyrosol		Dose: 498 µmol (Ileostomy volunteers) ** 24.7 ± 10.9 µmol 4.1 ± 3.1 µmol Dose: 526 µmol (healthy volunteers) * 21.6 ± 4.8 µmol 5.6 ± 8.8 µmol
[17]	Bioavailability assessment of oleuropein and HT from olive leaf extract, showing higher absorption, faster HT peak, and greater exposure in liquid form compared to capsules and male gender (n = 9)	Olive leaf extract (OLE) taken as capsule Olive leaf extract (OLE) taken as liquid	51.1 mg oleuropein, 9.7 mg HT 76.6 mg oleuropein, 14.5 mg HT 51.1 mg oleuropein, 9.7 mg HT 76.6 mg oleuropein, 14.5 mg HT	Sulphated derivatives of HT and HVA Sulphated derivatives of HT and HVA Sulphated derivatives of HT and HVA Sulphated derivatives of HT and HVA	90 ± 0 min 96 ± 13 min 53 ± 29 min 75 ± 17 min				
[40]	Dose-dependent HT bioavailability was hypothesized and confirmed HT-3S was the most abundant metabolite	HT-based product from olives, called Hytolive (HT, 10%)	5 mg HT 25 mg HT				HT HT-4-G HT-3-G HT-3-S HT-4-S HT HT-G-4 HT-G-3 HT-S-3 HT-S-4		Dose: 5 mg * 0 mg 0.11 mg 0.14 mg 1.18 mg (16.6%) 0.01 mg Dose: 25 mg 0 mg 0.46 mg 0.72 mg 4.15 mg (23.1%) 0.07 mg
[34]	Bioavailability study was performed with an enriched supplement with different doses. HT-3-S, and DOPAC were the main metabolites found in plasma and urine. (n = 12)	Two olive-derived watery supplements containing different doses	30.58 mg HT 61.48 mg HT	HT-3-S HVA DOPAC HT-3-S HVA DOPAC	30 min 30 min 30 min 30 min 30 min 30 min	384.96 ± 62.63 nmol/L 868.86 ± 111.86 nmol/L 301.07 ± 13.43 nmol/L 406.28 ± 32.68 nmol/L 948.76 ± 160.19 nmol/L 466 ± 147.68 nmol/L	HT-3-S HT-4-S HT-3-G HT-4-G HVA DOPAC HT-3-S HT-4-S HT-3-G HT-4-G HVA DOPAC		Dose: 30.58 mg * 16.58 ± 6.0 µmol 5.32 ± 16.23 µmol 0.46 ± 0.35 µmol 0.15 ± 0.10 µmol 36.1 ± 20.08 µmol 44.31 ± 3.93 µmol Dose: 61.48 mg 20.05 ± 1.55 µmol 0.16 ± 0.05 µmol 0.87 ± 0.13 µmol 0.41 ± 0.10 µmol 46.16 ± 5.37 µmol 59.74 ± 3.00 µmol

* Only total amounts were determined. ** Data referred to ileostomy effluent of 24 h.

**Table 3:** Human studies on hydroxytyrosol bioavailability from enriched food products.

Ref.	Overview	HT Source	Administered Dose	Plasma Presence Urine			Urine		
				Metabolites	Peak Time	Maximum Concentration	Metabolites	Peak Time	Total Amounts/Concentrations
[41]	Crossover study is realized, demonstrating HT (HT) in HT-enriched biscuits is highly bioavailable, with metabolites peaking within 0.5–1 h post-consumption, and significant reduction in postprandial oxidized-LDL levels. Major metabolites found were sulfated derivatives of HT and DOPAC	HT enriched biscuits and non-enriched ones	30 g biscuits (5.25 mg HT)	HT-G (2) DOPAC-G DOPAC HT-S DOPAC-S HVA-G HVA HVA-S	36 ± 13 min 60 ± 21 min 36 ± 13 min 47 ± 25 min 56 ± 23 min 66 ± 24 min 53 ± 24 min 53 ± 24 min	0.002 ± 0.001 µmol/L 0.009 ± 0.003 µmol/L 0.03 ± 0.01 µmol/L 1.0 ± 0.03 µmol/L 0.5 ± 0.1 µmol/L 0.007 ± 0.003 µmol/L 0.10 ± 0.02 µmol/L 0.07 ± 0.02 µmol/L	HT-G (1) HT-G (2) DOPAC-G DOPAC HT-S DOPAC-S HVA-G HVA HVA-S	0–3 h 0–3 h 12–24 h 0–3 h 0–3 h 0–3 h 0–3 h 0–3 h -	2.6 ± 0.8 ng * 3 ± 1 ng 98 ± 28 ng 18 ± 4 ng 2168 ± 547 ng 1457 ± 439 ng 28 ± 6 301 ± 48 n.d.
[18]	Demonstration of matrix-dependent bioavailability, with extra virgin olive oil identified as the optimal matrix HT peak was reached at 30 min. (n = 20)	HT added to flax oil HT added to grapeseed oil HT added to margarine HT added to pineapple juice	5 mg/20 g 5 mg/20 g 5 mg/20 g 5 mg/20 g	HT HT HT HT		n.d. n.d. n.d. n.d.	HT HT-Acetate DOPAC HvOH HT HT-Acetate DOPAC HvOH HT HT-Acetate DOPAC HvOH HT HT-Acetate DOPAC HvOH	0–8 h 0–8 h 0–8 h 0–8 h	~0.3 μg/mg creatinine ~20 μg/mg creatinine ~18 μg/mg creatinine ~30 μg/mg creatinine ~0.3 μg/mg creatinine ~20 μg/mg creatinine ~20 μg/mg creatinine ~20 μg/mg creatinine ~0.3 μg/mg creatinine ~40 μg/mg creatinine ~40 μg/mg creatinine ~5 μg/mg creatinine ~0.3 μg/mg creatinine ~20 μg/mg creatinine ~20 μg/mg creatinine ~30 μg/mg creatinine

* Amounts are obtained from the major excretion fraction. n.d. Not detected.

## Data Availability

The original contributions presented in this study are included in the article.
